# The emotion potential of simple sentences: additive or interactive effects of nouns and adjectives?

**DOI:** 10.3389/fpsyg.2015.01137

**Published:** 2015-08-11

**Authors:** Jana Lüdtke, Arthur M. Jacobs

**Affiliations:** ^1^Department of Education and Psychology, Experimental and Neurocognitive Psychology, Freie Universität BerlinBerlin, Germany; ^2^Department of Psychiatry and Psychotherapy, Charité Universitätsmedizin BerlinGermany; ^3^Languages of Emotion, Freie Universität BerlinBerlin, Germany; ^4^Dahlem Institute for Neuroimaging of EmotionBerlin, Germany

**Keywords:** sentence comprehension, affective sentence structure, emotional valence, supralexical evaluation, neurocognitive poetics model, affective congruency effect, sentence verification, situation model building

## Abstract

The vast majority of studies on affective processes in reading focus on single words. The most robust finding is a processing advantage for positively valenced words, which has been replicated in the rare studies investigating effects of affective features of words during sentence or story comprehension. Here we were interested in how the different valences of words in a sentence influence its processing and supralexical affective evaluation. Using a sentence verification task we investigated how comprehension of simple declarative sentences containing a noun and an adjective depends on the valences of both words. The results are in line with the assumed general processing advantage for positive words. We also observed a clear interaction effect, as can be expected from the affective priming literature: sentences with emotionally congruent words (e.g., The grandpa is clever) were verified faster than sentences containing emotionally incongruent words (e.g., The grandpa is lonely). The priming effect was most prominent for sentences with positive words suggesting that both, early processing as well as later meaning integration and situation model construction, is modulated by affective processing. In a second rating task we investigated how the emotion potential of supralexical units depends on word valence. The simplest hypothesis predicts that the supralexical affective structure is a linear combination of the valences of the nouns and adjectives (Bestgen, 1994). Overall, our results do not support this: The observed clear interaction effect on ratings indicate that especially negative adjectives dominated supralexical evaluation, i.e., a sort of negativity bias in sentence evaluation. Future models of sentence processing thus should take interactive affective effects into account.

## Introduction

In contrast to a comprehensive neurocognitive poetics model of literary reading (Jacobs, 2011, 2015a,b) most theories of word recognition and sentence processing disregard the role of affective content and emotional experiences. Nevertheless, there is much empirical evidence showing that at the three main levels of psychological description, i.e., experiential, behavioral, and neuronal, the processing of emotion-laden words differs from that of neutral words (for review: Citron, 2012; Jacobs et al., 2015, this issue). Despite differences in experimental designs and measures, “emotional” words are typically understood as words expressing emotions (e.g., sad, lonely, proud, jolly) or possessing “emotional connotations” (e.g., betrayer, nasty, family, successful). Such words are usually characterized within the framework of dimensional models of emotion along the two axes of arousal and valence. Studies on single word processing, which constitute the vast majority of research on affective text processing, have highlighted various processing differences for emotional compared to neutral words in various time windows following word (e.g., Kuchinke et al., 2005, 2007; Kissler et al., 2006, 2009; Herbert et al., 2008; Hofmann et al., 2009; Kousta et al., 2009; Schacht and Sommer, 2009; Scott et al., 2009; Palazova et al., 2011; Citron, 2012; Sheikh and Titone, 2013; Kuperman et al., 2014; Recio et al., 2014). In most studies differences are more pronounced for positive compared to negative words (Kuchinke et al., 2005, 2007; Kanske and Kotz, 2007; Estes and Verges, 2008; Larsen et al., 2008; Hofmann et al., 2009; Schacht and Sommer, 2009; Scott et al., 2009; Palazova et al., 2011; Recio et al., 2014).

## Affective word processing in context

In order to develop sufficiently precise neurocognitive models of affective word processing (Jacobs et al., 2015, this issue) such studies on single word processing appear to be in need of complementary research characterized by higher degrees of ecological validity, as shown in recent attempts at more natural approaches to language use and reading (e.g., Altmann et al., 2012; Hsu et al., 2014, 2015a,b,c; Wallot, 2014; Willems, 2015). Up to now only, a few studies have examined affective word processing in sentence contexts. Pioneers in this respect were Fischler and Bradley (2006), who investigated the processing of coherent adjective noun phrases. They basically replicated ERP differences between negative and positive words compared to neutral ones in different time windows, which were usually found in studies on single word processing. The enhanced processing for emotional compared to neutral words were also replicated for words embedded in whole meaningful sentences (De Pascalis et al., 2009; Holt et al., 2009; Bayer et al., 2010; Martín-Loeches et al., 2012; Scott et al., 2012; Delaney-Busch and Kuperberg, 2013; Ding et al., 2014). Other studies demonstrated that the pronounced effect for positive words could also be observed within phrases and sentences. For example, Schacht and Sommer (2009) examined the processing of positive, negative and neutral verbs in a minimal semantic context (i.e., word pairs) and found that the ERPs for emotional verbs following a single noun reflect enhanced processing compared to neutral ones. Moreover, their behavioral data showed an advantage for positive verbs across different tasks. Furthermore, Jiang et al. (2014) demonstrated that sentences containing a high pleasure adjective lead to shorter reaction times in a valence decision task than sentences containing low pleasure adjectives. This effect occurred irrespective of sentence polarity. Even when containing a negation, reaction times for sentences with high pleasure adjectives were shorter, although the negation changed the valence of the whole sentences. The behavioral effects were accompanied by an early ERP effect for the emotional adjectives indicating advanced processing for high pleasure adjectives. Besides the main effect of valence, the authors described a significant interaction between word valence and sentence polarity in later time windows, which were more strongly associated with contextual integration. They assumed that after the rapid extraction of word valence, further processing of emotional words like the integration in a mental representation of the whole phrase (e.g., a situation model), is influenced by the sentence context. There exists empirical evidence, that not only context information influences the processing of emotional word**s** but also that the emotional salience of a word can modulate its integration into sentences or discourse (León et al., 2010; Moreno and Vázquez, 2011; Leuthold et al., 2012; Ding et al., 2014; Hsu et al., 2014, 2015a,b,c). Wang et al. (2013) presented plausible question-answer pairs while varying the emotional salience of the target word and its linguistic focus. Besides typical early ERP effects indicating an initial highly automatic processing advantage of emotional words, an interaction between emotional salience and information structure in the later N400 component was observed. Corresponding with Jiang et al.'s (2014) results the authors interpreted this interaction as evidence for an attention-emotion interaction at later processing stages associated with the integration of the emotional meaning into the mental representation of the whole sentences (see also De Pascalis et al., 2009; Holt et al., 2009; Martín-Loeches et al., 2012). Whether the emotionality of words influences the processing of following neutral words was tested by Ding et al. (2014). They presented orthographically correct and incorrect neutral object nouns after emotional and neutral verbs in the context of simple declarative sentences. The ERP effects observed for the orthographic violation differed for emotional compared to neutral context. Whereas, after neutral verbs orthographically incorrect nouns elicited a smaller P2 and a larger N400 compared to correct nouns, only a late positive effect starting at 500 ms was observed after emotional verbs. The authors' interpretation was that emotional words captured and held more attentional processes compared to neutral ones and thus compromised the early processing of following neutral words leading to a general reanalysis especially on perceptual and lexico-semantic levels.

## Does the processing of emotional words influence the processing of other emotional words?

Despite the growing number of studies investigating effects of embedded emotional words, up to now is not clear to what extent the processing of such words influences the processing of other emotional words presented in the same sentence. Regarding the processing of single words effects of the emotional connotation of one word on the processing of a following word are usually described as affective priming (cf. Fazio, 2001; De Houwer et al., 2009). Since Fazio et al. (1986) first described a processing advantage for emotional target words following an emotionally congruent prime in an evaluative decision task, subsequent research replicated and extended the original findings many times (see Klauer and Musch, 2003, for a review). Typically, faster and less error-prone responses were observed when prime and target are affectively congruent (i.e., positive–positive, negative–negative) than when they are incongruent (i.e., positive–negative, negative–positive). Most important, affective priming effects have also been found for (implicit) tasks not focusing on the processing of the emotional meaning, e.g., naming and lexical decision (Hill and Kemp-Wheeler, 1989; Bargh et al., 1996; De Houwer and Randell, 2004; Spruyt et al., 2007). These studies support the view, that affective priming effects can be explained with a pre-activation of evaluatively congruent targets by spreading activation within a semantic network or by semantic pattern priming in a distributed memory system (Bargh et al., 1996; Fazio, 2001; Spruyt et al., 2007; see Hofmann and Jacobs, 2014; for a neurocomputational model implementing such a mechanism). Especially in evaluative decision tasks, processes related to response priming also seem relevant. Here it is assumed that affective primes automatically activate the corresponding evaluative response that is the correct one in congruent, but the incorrect one in incongruent trials (Klauer and Musch, 2003). Klauer et al. (2005) assumed that response-related priming should be larger than semantically mediated priming effects. This is in line with the observation that in tasks in which the affective prime-target congruency is unrelated to the response and the task at hand, affective priming effects could not be observed reliably (Klauer and Musch, 2003). Primarily due to the last fact it still remains unclear, whether or not the processing of emotional words is mutually interrelated when they are embedded in sentences. Ding et al. (2014) demonstrated that emotional words influenced the processing of upcoming neutral words. Whether these influences differ for upcoming emotional words as suggested by semantically mediated affective priming effects reported for single word processing is an empirical question. Up to now only Fischler and Bradley's (2006) study explored possible interaction effects. They reported no significant congruence effects on ERPs recorded for positive, neutral, and negative nouns following positive, neutral, or negative adjectives. The ERPs observed for the nouns did not differ as a function of the emotional meaning of the preceding adjective, irrespective of whether the two consecutive words were processed as phrases or as single words. As discussed by the authors, one reason for the non-occurrence of any congruence effects could be the presentation mode, i.e., the ERP-typical serial presentation of words separated by a blank monitor. As shown by Hermans et al. (2001) affective priming effects are moderated by the stimulus onset asynchrony (SOA) between prime and target. They appear to be based on fast-acting automatic processes, are quite short-lived, and thus should only be observed reliably for SOAs below 300 ms. Fischler and Bradley, however, used longer SOAs of 750 ms between the emotional words. As discussed by the authors these circumstances might have minimized the possibility to observe any interaction.

In spite of the possibility that affective priming is limited to tasks focusing on valence evaluation (Klauer and Musch, 2003), results from a recent study on sentence comprehension indicated, that the processing of emotional words embedded in sentences could be influenced by emotional information delivered by preceding words. León et al. (2010) measured ERPs for positive or negative adjectives qualifying the emotion of protagonists that were introduced in narratives describing emotional episodes presented before the critical sentences. The protagonist's emotions mentioned in the critical sentences were either consistent or inconsistent with the preceding story. Inconsistent emotions were found to elicit larger N1/P2 and N400 complexes than consistent emotions, indicating clear interactions between the emotional valence of the critical word and the emotional meaning of the context. Given the significant temporal interval between the reading of the context story and the on-line sentence processing, the authors interpreted the congruency effects as a discourse level phenomenon. Although the authors argued, that such effects could not be observed in isolated words. It is unclear whether these effects could also be due to some form of long-term affective priming based on an automatic spread of evaluative activation (e.g., Eder et al., 2012).

## Affective meaning making at the supralexical level

Most of the studies presented above focus on the early processing of emotional words or its integration into sentence context. The issue of how the affective meaning of phrases or sentences as supralexical units is constructed from the words constituting this unit was not explored. Based on the logico-philosophical tradition since Frege, according to which the literal meaning of a sentence could be determined by the meanings of its parts and their syntactical combination, it can be assumed that the emotion potential of supralexical units is a (linear or non-linear) function of the emotion potential of the words included therein (Hsu et al., 2015c; Jacobs, 2015a,b). Accordingly, the simplest model predicting the emotion potential of a sentence should take into account only the emotional or connotative meaning of its component words while neglecting other potentially relevant influences like their syntactic role or the constituents' order (Jacobs, 2015a). Following this account a simple declarative sentence containing a positive noun and a negative adjective like “*The mother is bad*” should —on average—be evaluated as neutral. First empirical evidence for this “null-model” of supralexical affective meaning was obtained by Bestgen (1994) and Whissell (1994), both demonstrating that the valence of supralexical units could be predicted—to a considerable extent—as a function of the emotional or connotative meaning of their component words.

This most simple model does not take into account other potentially relevant variables, e.g., different syntactic roles of words, which could also be relevant for affective meaning construction. The simple “*The mother is bad*” contains a noun and an adjective. While nouns occur as the head of a noun phrase and refer to concrete entities such as people or things, adjectives characterize noun phrases and modify their meaning. Therefore, especially evaluative and emotive adjectives denoting specific features of possible noun referents may induce deeper elaborative affective processing than nouns. Empirical evidence for this assumption comes from an ERP-study by Palazova et al. (2011) reporting differences in emotional effects in single word processing of nouns and adjectives. Besides an early effect for emotional compared to neutral words for both nouns and adjectives, Palazova et al. observed a second emotion effect around 450 ms, but this time only for adjectives. They interpreted this Late Positivity Complex (LPC) observed for positive compared to both neutral and negative adjectives as an index of sustained and elaborate processing of the emotional aspects of adjectives, which was possibly not induced by nouns. Thus, if an adjective is presented not in isolation but as predicative adjective modulating the meaning of a noun, it can be assumed, that the emotional meaning of the adjective dominates the affective meaning of the supralexical unit as a whole. A recent study by Liu et al. (2013) demonstrated such a dominance effect for emotional adjectives. They compared valence evaluations for positive, neutral, and negative nouns, which were read after a positive or negative adjective or even in a non-context condition without a preceding word. Although the participants evaluated only the emotional valence of the noun, preceding emotional adjectives modulated the results. Positive adjectives biased the noun evaluation toward stronger positive ratings compared to evaluations for isolated nouns whereas negative adjectives led to stronger negative noun evaluations compared to the non-context condition. The modulation effect was greatest when the preceding adjective was negative and the to-be-evaluated noun was positive. This superiority effect for negative adjectives is in line with the often observed *negativity bias* describing the stronger impact of negatively valenced compared to positively valenced events on different evaluation and attention related processes (for an overview see Baumeister et al., 2001). It is assumed, that the negativity bias operates especially at the evaluative-categorization stage (Ito et al., 1998) and that negative information therefore dominates the evaluation of combinations of negative and positive entities yielding more negative evaluations than the algebraic sum of individual valences would predict (Rozin and Royzman, 2001). Taken together, the assumed dominance of emotional adjectives in the evaluation of simple supralexical units and the ubiquitous negativity bias lead to the prediction, that valence ratings of simple declarative sentences like “*The mother is bad”* should be characterized by a negativity bias especially for adjectives. Challenging the simple null model outlined above, this prediction includes an interactivity assumption of affective word processing.

## Aims of the present study

The present study was designed to investigate to what extent the processing of emotional words within a sentence context shows interactive effects. It is now a well-established result that both the early processing of a word as well as the following integration into the context of a phrase, sentence, or short discourse can be modulated by the emotional connotation of that word. Whether or not the processing of an emotional word is also influenced by the processing of other emotional words presented in the same sentence remains, however, an open question. Results from the field of affective priming suggest that interactive effects are possible even in tasks not focusing on the emotional meaning of the words or phrases. However, possible congruency effects are very short-lived. To observe reliable interaction effects, it seems necessary that the crucial words are processed within a time window of about 300 ms. In order to test such interaction effects between emotional words we therefore presented simple declarative sentences containing a noun and an adjective (e.g., The grandpa is clever) separated only by a short auxiliary verb. Combined with a self-paced reading paradigm this ensured that the critical time window was obtained. To investigate the influence of the emotional connotation the valence of the nouns and adjectives was manipulated using the Berlin Affective Word List (BAWL; Võ et al., 2009; Jacobs et al., this issue). To ensure processing at sentence level participants had to decide whether a sentence was meaningful (e.g., The grandpa is clever) or not (e.g., The cheese is intelligent).

Besides the question whether the early processing of emotional words presented in sentences is interrelated, we were also interested in seeing how the affective meaning of single words influenced the interpretation of the sentences as a supralexical unit. To investigate this aspect of meaning construction, we expand the present study by a second task requiring an explicit and deep processing of the affective meaning of words. The meaningful emotional sentences used in the sentence verification task were presented again, but this time participants had to rate the emotional valence and arousal of the whole sentences as supralexical units.

## Hypotheses

Although a sentence verification task does not require explicit processing of the affective meaning of words to yield correct responses, we generally expected that the emotional valence of nouns and adjectives automatically influences the reaction times. Empirical research on single word processing provides ample demonstrations that even a superficial semantic elaboration as required for lexical decisions is sufficient for observing emotional effects (Jacobs et al., 2015, this issue). Based on the above-mentioned literature, we therefore anticipated shorter verification times for sentences containing emotional adjectives compared to sentences with neutral ones. Moreover, due to the often-reported processing advantage of positive over negative words, we also expected shorter verification times for sentences with positive compared to negative adjectives and nouns, i.e., a positivity superiority effect. If the emotionality of a word basically influenced its early processing (e.g., Recio et al., 2014)—and if the positivity superiority was a general phenomenon—the following rank order of verification times should be obtained: emotionally congruent sentences with no positive word > emotionally incongruent sentences with only one positive word > emotionally congruent sentences with two positive words. If, on the other hand, the processing of emotional nouns and adjectives interacted, as suggested by the affective priming literature, verification times should be faster for sentences with emotionally congruent words (e.g., sentences with positive nouns followed by positive adjectives) than for emotionally incongruent sentences and sentences with neutral adjectives. This rank order should be reflected in a significant interaction between the two variables indicating the valence of the noun (valence group—noun) and the adjective (valence group—adjective). To ensure that a potential interaction effect depends primarily on the emotional relation between nouns and adjectives, we controlled the semantic associations between them. As shown neurocomputationally by Hofmann and Jacobs (2014) direct first-order co-occurrences of two words are valid indices of their semantic association. We thus matched the sentence based first-order co-occurrences for nouns and adjectives in all six experimental conditions.

For the evaluation task the simple “null-model” presented above predicts that the valence ratings conducted in the second part of the study should depend equally on the valence of both of their constituents, the noun as well as the adjective. More precisely, lower valence ratings should be observed for sentences with negative compared to positive adjectives and nouns. This should lead to the following rank order of the sentence valence ratings: sentences with two negative words < sentences with only one negative word < sentences with only one positive word < sentences with two positive words.

Taking into account other potentially relevant variables especially the syntactic role, different predictions arise. The assumed dominance of emotional adjectives in the evaluation of simple supralexical units and the ubiquitous negativity bias would predict that the valence ratings of our simple declarative sentences show a negativity bias especially for adjectives. We thus hypothesized that sentences with a negative adjective were evaluated as strongly negative, while the valence of the nouns should have only a minor influence. For sentences with neutral and positive adjectives, the overall valence evaluation should also be influenced by both the emotional connotation of the adjectives and the nouns. If these adjectives were preceded by negative nouns, the overall valence ratings should be biased in a more negative direction compared to sentences with positive nouns.

## Methods

### Participants

Thirty-six participants (21 female; age: *M* = 28.36, *SD* = 6.93, range = 20–47), all native German speakers, were recruited at the Freie Universität Berlin. All had normal or corrected-to-normal vision and were paid for participation. The whole experiment followed the rules set by the ethical guidelines of the German Psychological Society's 121 (DGPs, 2004, CIII). Participants were informed about taking part in research, about the possibility of quitting the experiment with no disadvantage at any time and about the fact that all data was anonymously collected and analyzed. They provided informed consent and allowed us to use their collected data anonymously for publications.

### Stimuli

Ninety-six item sets were constructed, each containing six different meaningful declarative sentences. Each of the resulting 576 different experimental sentences contained a noun in the subject position, the auxiliary verb *be*, and an adjective describing a feature of the noun. To construct the different sentences, we selected a positive and a negative noun as well as a positive, a neutral, and a negative adjective for each item set from the extended version of the BAWL-Reloaded (Conrad et al., unpublished) which included valence ratings from a seven-point rating scale (reaching from −3 to 3) for over 6000 words. The selection was based on the following criteria: (1) mean rating of emotional valence in one of three emotional valence categories—negative (mean rating <-1.0), neutral (−0.5 < mean rating < 0.5), or positive (mean rating >1.0); (2) no differences due to the standard deviation of single word ratings between groups of positive and negative words, and (3) the combination of the positive and the negative noun with the positive, neutral, and negative adjective should yield six different meaningful sentences per item set. Table 1 gives examples for the six combinations in one item set. Table 2 reports means and standard deviations for important features of the words used in the emotional valence categories of nouns and adjectives.

**Table 1:** **Example sentences and mean co-occurrence measures (Ms and SDs) for each of the six conditions**.

**Version**	**Example sentences**	**Co-occurrence**	
		***M***	***SD***
Positive noun–Positive adjective	The grandpa is clever	0.48	2.82
Positive noun–Neutral adjective	The grandpa is small	2.40	24.56
Positive noun–Negative adjective	The grandpa is lonely	0.20	1.51
Negative noun–Positive adjective	The burglar is clever	0.87	4.41
Negative noun–Neutral adjective	The burglar is small	1.15	4.85
Negative noun–Negative adjective	The burglar is lonely	0.21	2.57
Nonsense sentences	The milk is careful	–	–

*Sentences based co-occurrence measures were taken from the German corpus of the “Wortschatz” project (http://corpora.informatik.uni-leipzig.de/, status: December 2006; Quasthoff et al., 2006)*.

**Table 2:** **Stimulus characteristics (Ms and SDs)**.

	**Nouns**				**Adjectives**					
	**Positive**		**Negative**		**Positive**		**Neutral**		**Negative**	
	***M***	***SD***	***M***	***SD***	***M***	***SD***	***M***	***SD***	***M***	***SD***
Word frequency^a^	1332.05	2347.98	1514.15	3477.93	909.42	1037.85	1395.59	4112.58	951.12	3335.95
Mean Valence^b^	1.56	0.41	−1.77	0.56	1.74	0.47	0.03	0.28	−1.82	0.39
SD Valence^b^	1.01	0.20	0.96	0.25	0.86	0.26	0.83	0.21	1.05	0.25
Mean Arousal^b^	2.75	0.67	3.55	0.65	2.73	0.58	2.72	0.63	3.26	0.58
Imageability^b^	5.10	1.03	4.97	0.86	3.55	0.90	3.54	0.95	3.50	0.85
Number of letters	7.09	2.25	7.32	2.12	7.93	2.14	7.50	2.17	7.71	2.20
Number of syllables	2.34	0.79	2.36	0.85	2.36	0.74	2.33	0.83	2.34	0.82

a *Word frequencies were taken from the dlexDB database (Heister et al., 2011)*.

b *Ratings were taken from the extended version of the Berlin Affective Word List–Reloaded (Conrad et al., unpublished)*.

Nouns in the two emotional valence categories did not differ significantly in mean frequency [*t*_(95)_ = −0.42, *p* = 0.68], number of letters [*t*_(95)_ = −0.83, *p* = 0.41], number of syllables [*t*_(95)_ = −0.19, *p* = 0.85], mean imageability ratings [*t*_(86)_ = 0.55, *p* = 0.58], and standard deviations for valence ratings [*t*_(71)_ = −1.43, *p* = 0.16]. In line with our selection criteria, the mean valence ratings for the nouns differed significantly [*t*_(95)_ = 50.12, *p* < 0.0001]. Due to the typical asymmetrical inverted U-shaped relation between valence and arousal ratings of the BAWL (e.g., Võ et al., 2009), the mean arousal ratings for the group of positive and negative nouns also differed significantly [*t*_(93)_ = −8.71, *p* < 0.001]. Adjectives in the three different emotional valence categories did not differ significantly in mean frequency [*F*_(2, 94)_ = -0.64, *p* = 0.53], number of letters [*F*_(2, 94)_ = 0.88, *p* = 0.42], number of syllables [*F*_(2, 94)_ = 0.04, *p* = 0.96], and mean imageability ratings [*F*_(2, 74)_ = 0.01, *p* = 0.99]. The standard deviations of the valence ratings were equal for positive and negative adjectives [*t*_(62)_ = 1.83, *p* = 0.14], whereas neutral adjectives had significantly higher standard deviations [negative vs. neutral: *t*_(69)_ = −3.33, *p* = 0.001; positive vs. neutral: *t*_(69)_ = −5.16, *p* < 0.001]. As for the nouns, mean valence ratings differed across the three emotional valence categories [positive vs. negative: *t*_(95)_ = 55.84, *p* < 0.0001; positive vs. neutral: *t*_(95)_ = 29.77, *p* < 0.0001; negative vs. neutral: *t*_(95)_ = −33.81, *p* < 0.0001]. The arousal ratings differed between positive and negative adjectives [*t*_(95)_ = −6.67, *p* < 0.0001], and neutral vs. negative adjectives [*t*_(95)_ = 6.14, *p* < 0.0001], but not for positive vs. neutral adjectives [*t*_(95)_ = 0.09, *p* = 0.93]. To control for different semantic relations between nouns and adjectives in the sentences of each condition, sentences based co-occurrence measures were collected from the German Corpus of the “Wortschatz” project (http://corpora.informatik.uni-leipzig.de/, status: December 2006; Quasthoff et al., 2006). There were no significant differences between the six conditions [*F*_(5, 567_) = 0.60, *p* = 0.70; all pairwise comparisons using Tukey's honestly significant difference tests were also not significant (all *p* > 0.69)]. Moreover, the mean sentence based co-occurrences (see Table 1) indicated no significant semantic associations between nouns and adjectives in most sentences: Only in 4% of all sentences, the co-occurrence measures exceeded the critical value indicating a semantic association (Hofmann et al., 2011). Ninety-six additional nonsense filler sentences were constructed. All of them followed the structure of the meaningful declarative sentences with the exception that nonsense “meanings” were generated by combining animated nouns with adjectives describing features of inanimated objects or vice versa (e.g., The milk is careful).

### Design and procedure

The study was divided into two parts: a sentence verification task followed by a rating task. In both parts each participant read only one of the six sentences of an item set. The 96 experimental item sets were assigned to six groups, the 36 participants to six groups, and the assignment of versions to both groups followed a 6 × 6 × 6 Latin square. We employed a 2 (valence group–noun) × 3(valence group–adjective) design with both variables being manipulated within participants and item sets. Each participant verified 16 sentences in each of the six conditions. Each of the six sentences per item set were verified by eight participants. The sentences used in the verification task were presented again in the rating task. To prevent fatigue and increase the reliability and ecological validity of the evaluations, the participants rated only half of the experimental stimuli. We therefore divided each of the six participant groups further into two subgroups. One subgroup rated the first half of the experimental sentences for this participant group, the other subgroup rated the second half. Each participant evaluated eight sentences per condition and each sentences was rated by four participants.

In the sentence verification task, each trial started with the presentation of a blank monitor for 1000 ms, followed by a fixation cross in the center of the screen for 800 ms. After presenting an additional blank screen for 800 ms, a sentence appeared in the center with black letters on white background (in 20 point Arial font). All sentences appeared in one line. The participants were instructed to decide as quickly and accurately as possible whether the presented sentences made sense or not. They indicated their responses with pressing the left and the right arrow key with the left and right index finger. Upon response registration, the sentence disappeared. During the experimental trials participants were not given any feedback on their responses. However, the verification task started with 10 practice trials, which included feedback. Then the 96 experimental sentences were presented in random order intermixed with the 96 nonsense filler sentences. We used a complete randomization to make sentence order individual for each participant.

The sentence ratings started with two examples. Emotional valence was rated on a nine-point scale ranging from very negative (−3) to neutral to very positive (+3). In addition to the verbal anchor, the valence scale of the Self-Assessment Manikin (SAM; Bradley and Lang, 1994) was presented. Arousal was rated on a five-point scale ranging from 1 [ruhig (very calm)] to 5 [aufregend (exciting)], again using the corresponding SAM-scale (cf. Schmidtke et al., 2014). Each trial in the rating part started with the presentation of a sentence at the top of the screen together with the valence scale. Participants used the numbers of the keyboard to indicate their response. Then the arousal scale appeared below. Both ratings and reaction times were recorded. An experimental session took approximately 35 min.

### Analysis

All analyses are based only on the results of the experimental sentences. Results for filler sentences were discarded. Following recent recommendations for psycholinguistic experiments, statistical analyses were conducted using linear mixed effects regression models (e.g., Baayen, 2008; Baayen et al., 2008; Jaeger, 2008). These were run in R version 3.10 (R Core team, 2014) employing models with crossed effects of subjects and item sets using the lme4 package (Bates et al., 2014). For the analysis of verification times, as well as valence and arousal ratings of the whole sentences, the fixed effects in the models included the categorical variables valence group-noun (VG-N: positive vs. negative) and valence group-adjective (VG-A: negative vs. neutral vs. positive). Moreover, due to the reported arousal differences between valence categories, the arousal ratings for nouns (ARO-N) and adjectives (ARO-A) taken from the extended BAWL (Conrad et al., unpublished) were included as metrical covariates. To avoid collinearity and maximize likelihood of model convergence, both variables were centered prior to analysis (Baayen, 2008). Fixed effects were checked with Wald F-tests with a Kenward–Roger approximation of degrees of freedom. Random intercepts were included for subjects and item sets with, if possible, maximal random slopes (Barr et al., 2013). Error rates were analyzed using a logistic linking function (Jaeger, 2008). For the sake of conciseness, only significant tests associated with fixed effects are reported, as these are directly relevant to our hypotheses.

To further test our hypotheses, single contrasts based on the glht-function of the multicomp packages of R (Hothorn et al., 2008) were calculated for verification times and sentence evaluation ratings. To test the assumption of an affective priming effect in the sentence verification task, we first compared verification times of both types of emotional congruent sentences (sentences with only positive or only negative words) with those for both types of incongruent ones (positive nouns followed by negative adjectives and vice versa). Afterwards, further single contrasts were calculated to test the affective priming for positive and negative adjectives. A direct comparison of the priming effects for positive vs. negative adjectives was done with the testInteraction-function of the phia package (De Rosario-Martinez, 2013). Both the test of single contrasts as well as the testInteraction-function were also used to analyse the valence ratings of the evaluation task.

## Results

A first analysis of the accuracy data from the verification task showed that six of the 576 experimental sentences were falsely verified by more than 80% of the participants. These were excluded from further analysis. After the elimination, mean accuracy in the verification task was 94.53% (*SD* = 2.75) for the experimental sentences and 92.07% (*SD* = 5.18) for the filler sentences.

### Sentence verification

To analyze the accuracy data, logistic linking functions with random intercepts for subjects and item sets were calculated. To test the fixed effects of the two categorical Variables VG-N and VG-A, their interaction, and the two covariates ARO-N and ARO-A, Wald-Chi-squared statistics were calculated. We observed neither significant main effects for the two categorical variables VG-N (χ^2^ = 1.81, *p* = 0.18) and VG-A (χ^2^ = 4.61, *p* = 0.10), nor a significant interaction effect between them (χ^2^ = 2.34, *p* = 0.31), and also no significant main effects for the covariates (ARO-N: χ^2^ = 2.00, *p* = 0.16; ARO-A: χ^2^ = 0.48, *p* = 0.49). Hence, the plausibility of the experimental sentences appears identical for all six conditions.

Only correct responses were included in the analysis of verification times. After eliminating extreme verification times of over 15,000 ms, response times more than 3 standard deviations above a participant's and items mean were excluded (0.38% of correct responses). Because of a rightward skewed distribution of verification times, the Box–Cox transformation test was conducted to identify an optimal transformation to improve normality of distribution (Box and Cox, 1964). The test strongly suggests that reciprocal RTs but not log transformation are in a metric compatible with the normal-distribution assumption. Therefore, the linear mixed models were performed on 1/RT transformed verification times. We repeated the LMM analyses reported below using the untransformed response times instead of the reciprocal transformation and found essentially the same results.

In a first step, the appropriate random effect structure was tested starting with a model containing a maximum random effects structure with by-subject and by-item set intercepts, as well as by-subject and by-item set slopes for VG-A, VG-N, their interaction, and the covariates ARO-N and ARO-A (Barr et al., 2013). A stepwise elimination of the slopes was combined with a comparison of the fit of the model with and without this random effect based on the R-function ANOVA (Crawley, 2007) applying a chi-square test. If the removal of one slope caused no significant difference, the random effect was eliminated. At the end, the analysis of the fixed effects was done with a model including both intercepts and the by-item set slopes for VG-A and VG-N.

**Figure 1:**
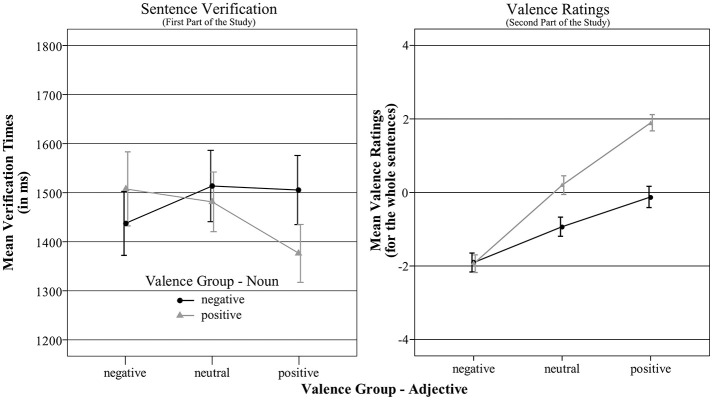
**Mean verification times and mean valence ratings for the whole sentences. Error bars show the ***SE*** of the mean for illustrative purposes**.

Estimates of the fixed effects based on effect coding for the two categorical predictors are reported in Table 3. The analysis yielded significant main effects for the factors VG-N [*F*_(1, 120.65)_ = 4.69, *p* = 0.03] and VG-A [*F*_(1, 101.80)_ = 3.93, *p* = 0.02]. In line with the hypothesized positivity superiority effect verification times of sentences with positive nouns (*M* = 1454.54, *SD* = 792.82) were shorter than those to sentences with negative nouns (*M* = 1485.562, *SD* = 792.82). Moreover, sentences containing a positive adjective (*M* = 1440.58, *SD* = 751.59) were also verified faster than sentences with neutral (*M* = 1497.46, *SD* = 758.43), and negative adjectives (*M* = 1473.01, *SD* = 808.46). Pairwise comparisons with Tukey's contrast showed that only the difference between sentences with positive vs. neutral adjectives was significant (*z* = 2.76, *p* = 0.02). The differences between sentences with positive and negative adjectives (*z* = 1.71, *p* = 0.20), and with neutral and negative adjectives (*z* = −1.04, *p* = 0.55) were not significant. The estimates for the two metrical covariates ARO-N and ARO-A indicated slightly positive relationships, but both effects were only marginally significant [ARO-N: *F*_(1, 178.13)_ = 3.51, *p* = 0.06; ARO-A: *F*_(1, 263.18)_ = 2.87, *p* = 0.09]. Most important, there was a highly significant interaction effect between the categorical variables VG-N and VG-A, as illustrated in Figure 1. The biggest differences occurred for sentences containing positive adjectives. Sentences were verified faster when positive adjectives were read after positive nouns (*M* = 1376.18, *SD* = 811.71) than after negative ones (*M* = 1505.35, *SD* = 681.30). Sentences with neutral adjectives following positive (*M* = 1481.26, *SD* = 687.02) nouns were verified slightly faster than sentences with neutral adjectives after negative nouns (*M* = 1513.56, *SD* = 823.67). For sentences containing negative adjectives, a reverse effect was observed. They were verified faster when the negative adjectives were read after negative nouns (*M* = 1437.27, *SD* = 739.31) than after positive ones (*M* = 1507.68, *SD* = 869.59).

**Table 3:** **LMM estimates of fixed effects for verification times, valence ratings, and arousal ratings**.

	**Verification times^a^**			**Valence ratings^b^**			**Arousal ratings^b^**		
	**Estimates**	**Std. error**	***t***	**Estimates**	**Std. error**	***t***	**Estimates**	**Std. error**	***t***
Intercept	7.96 × 10^−4^	3.30 × 10^−5^	24.11	2.03	0.04	57.35	1.57	0.03	59.90
VG-N^*^	−0.13 × 10^−4^	0.59 × 10^−5^	−2.13	−0.13	0.02	−8.05	0.001	0.01	0.20
VG-A_1_ (positive vs. negative)^*^	−0.02 × 10^−4^	0.71 × 10^−5^	−0.34	0.38	0.04	−10.42	0.04	0.02	2.23
VG-A_2_(positive vs. neutral)^*^	−0.16 × 10^−4^	0.72 × 10^−5^	−2.15	0.04	0.02	2.49	−0.02	0.01	−1.26
ARO-N	0.16 × 10^−4^	0.84 × 10^−5^	1.95	−0.003	0.02	0.48	0.08	0.02	4.00
ARO-A	0.15 × 10^−4^	0.87 × 10^−5^	1.77	0.01	0.02	−0.12	0.11	0.02	5.53
VG-N ^*^ VG-A_1_	0.12 × 10^−4^	0.47 × 10^−5^	2.48	0.13	0.02	6.44	−0.03	0.01	−2.33
VG-N ^*^ VG-A_2_	0.08 × 10^−4^	0.47 × 10^−5^	1.77	−0.02	0.02	−1.00	0.002	0.01	0.18

* *Effect coding was used for the categorical predictors VG-N and VG-A. Factor VG-A has three factor levels. Therefore, two fixed effects were reported. We called them VG-A_1_ and VG-A_2_*.

a *Verification times were 1/RT transformed. As random effects were included the intercepts for item set and subject, together with by-item set slopes for VG-N, VG-A_1_, and VG-A_2_*.

b *Valence ratings and arousal ratings were squared transformed. As random effects were included the intercepts for item set and subject, together with by-subject slopes for VG-A_1_ and VG-A_2_, and by-item set slopes for VG-N,VG-A_1_, VG-A_2_, and the interactions between VG-N and VG-A*.

To test the assumption of affective priming verification times of both types of emotional congruent sentences were compared with those for both types of incongruent ones. Emotionally congruent sentences were verified significantly faster than emotionally incongruent ones (*z* = −2.66, *p* = 0.008). Further contrasts showed that the priming effect occurred only for sentences with positive adjectives (*z* = 4.37, *p* < 0.0001) indicating emotional priming for positive adjectives after positive nouns. Although the described differences for negative adjectives were also compatible with an emotional priming effect after an emotional congruent noun, the difference was not significant (*z* = −0.13, *p* = 0.90). Moreover, the affective priming effect for positive adjectives was significantly stronger than that for negative adjectives (χ^2^ = −15.41, *p* < 0.0001). The difference observed between both types of sentences with neutral adjectives was not significant (*z* = 0.55, *p* = 0.58). To test whether the observed emotional priming effect for positive adjectives after positive nouns indicated a processing advantage, we compared verification times for sentences with positive adjectives after positive nouns to those for sentences with neutral adjectives after positive nouns. Again, this difference was significant (*z* = −4.23, *p* < 0.0001).

### Valence ratings

Since valence ratings for the whole sentences were not normally distributed, analyses were performed on squared-transformed values as indicated by the Box–Cox transformation test (Box and Cox, 1964). Again, the first step was the identification of the appropriate random effect structure starting with a model containing a maximum random effects structure with by-subject and by-item set intercepts, as well as by-subject and by item set slopes for VG-A, VG-N, their interaction and the covariates ARO-N and ARO-A (Barr et al., 2013). The stepwise elimination of the slopes together with a comparison of the fit of the model with and without this random effect indicated that only the by-subject slope for VG-A and the by-item set slopes for VG-N, VG-A as well as the interaction between both should be included in the analysis of the fixed effects.

Estimates of the fixed effects based on effect coding for the two categorical predictors are reported in Table 3. There was a significant main effect for the factor VG-N [*F*_(1, 119.11)_ = 61.63, *p* < 0.0001], indicating lower valence ratings for sentences with negative (*M* = −0.98, *SD* = 2.38) compared to positive nouns (*M* = 0.06, *SD* = 2.56). The main effect for VG-A was also significant [*F*_(1, 35.27)_ = 73.97, *p* < 0.0001]. Sentences with negative adjectives (*M* = −1.92, *SD* = 2.10) yielded lower ratings than sentences with neutral ones (*M* = −0.36, *SD* = 2.22) which were rated lower than sentences with positive adjectives (*M* = 0.89, *SD* = 2.40). Pairwise comparisons with Tukey's contrast showed that all differences were significant (*z* > 7.80, *p* < 0.0001). The covariates ARO-N [*F*_(1, 265.33)_ = 0.20, *p* = 0.65] and ARO-A [*F*_(1, 175.34)_ = 0.01, *p* = 0.91] had no predictive power. Again, as for verification times, there was a highly significant interaction between VG-N and VG-A [*F*_(1, 88.81)_ = 21.72, *p* < 0.0001], illustrated in Figure 1. Sentences with negative adjectives following negative nouns (*M* = −1.91, *SD* = 2.17) were rated as negative as sentences with negative adjectives after positive nouns (*M* = −1.94, *SD* = 2.03; single contrast: *z* = 0.07, *p* = 0.95). Sentences with neutral adjectives after positive nouns were rated significantly more positive (*M* = 0.20, *SD* = 2.13) than sentences with neutral adjectives after negative nouns (*M* = −0.94, *SD* = 2.17; single contrast: *z* = 6.38, *p* < 0.0001). The same pattern was observed for sentences with positive adjectives. Ratings were higher for sentences with positive adjectives after positive (*M* = 1.91, *SD* = 1.87) than after negative nouns (*M* = −0.12, *SD* = 2.45; single contrast: *z* = 9.05, *p* < 0.0001). The differences between the two sentence types with positive adjectives were stronger than those between the two types of sentences with neutral adjectives (χ^2^ = 9.14, *p* = 0.003).

## Discussion

We presented simple declarative sentences with positive and negative nouns followed by either positive, neutral, or negative adjectives to test whether the processing of emotional words embedded in a sentence context is interactive. In part I of the study, participants read the sentences and decided as quickly as possible whether they were meaningful. In part II they read half of the sentences again and rated the valence and arousal of the sentences as supralexical units.

The verification task yielded three main results. First, we replicated the positivity superiority effect often observed in single word processing (e.g., Hofmann et al., 2009; Citron, 2012) and in EEG studies exploring emotional effects in sentences processing (e.g., Fischler and Bradley, 2006; Bayer et al., 2010; Ding et al., 2014; Jiang et al., 2014). This was indicated by significant main effects for VG-N and VG-A. Second, we observed a significant interaction between these two variables. Third, single contrasts showed that shorter verification times for sentences with emotional compatible words were observed only for positive words. In the following, these effects are discussed in more detail.

Although a priori the sentence verification task does not require processing of the affective meaning of words to yield correct responses, we replicated the positivity superiority effect indicating a clear processing advantage for sentences with positive words compared to sentences with negative and/or neutral words. The assumed enhanced attention allocation for emotional compared to neutral words, and especially for positive words compared to neutral and negative ones, likely facilitated their early processing and the subsequent meaning-based decisions. When sentences contained a neutral or a negative word, no facilitation occurred and participants needed more time to decide about the sentences' meaningfulness.

In contrast to Fischler and Bradley (2006), we observed a clear processing advantage for emotionally congruent sentences. Verification times for sentences with words from the same valence category (e.g., positive adjectives after positive nouns) were shorter than verification times for sentences with words from different valence categories (e.g., positive adjectives after negative nouns). This result corresponds with affective priming effects reported for single word processing (cf. Fazio, 2001; Klauer and Musch, 2003) and also with the assumed discourse dependent congruency effect reported by León et al. (2010). Two mechanisms can be hypothesized to explain the observed interaction, a first one operating at the lexical level, and a second one at the supralexical level. At the lexical level, the benefit for congruent sentences may be related to an automatic spread of semantic activation (Hofmann et al., 2011; Eder et al., 2012; Hofmann and Jacobs, 2014). Different studies have convincingly demonstrated that such processes operating at an early encoding level contribute to the affective priming effect (e.g., Spruyt et al., 2007). Although sentence verification does not require explicit processing of affective word meanings, the observed *positivity superiority effect* supports the assumption that participants automatically processed affective stimulus dimensions, as is also known from lexical decision studies. Moreover, the short distance between nouns and adjectives provided a quasi-optimal condition for observing short-lived effect of preactivating memory representations of affectively related words (Hermans et al., 2001). When an emotional noun was followed by an emotionally congruent adjective, spreading activation presumably facilitated its early processing.

At the supralexical level, the emotional congruency between noun and adjective could also facilitate the integration of the emotional words in a meaningful mental representation of the described state of affairs, i.e., a situation model. Some authors have interpreted the larger N400 response to affectively incongruent trials in standard affective priming paradigms in terms of integration difficulties of affective information in incongruent conditions (Eder et al., 2012). However, empirical studies on context or discourse related integration effects of affective congruency are rare. León et al. (2010), for example, observed an early automatic ERP response (N100/P200), followed by a later discourse-level N400 for emotionally incongruent sentences. Whether or not the observed ERP are related to prolonged reading times was not measured. Behavioral studies on elaborative inferences about the emotional states of story protagonists reported shorter reading times for emotional congruent compared to incongruent sentences (cf. Gernsbacher et al., 1992; de Vega et al., 1996). The authors interpeted the prolonged reading times, at least in part, in terms of integration difficulities during situation model construction and updating. Because such integration is also a necessary step for understanding and verifying single sentences, we propose that the interaction observed in our study is based on both faster early processing and facilitated later integration in the congruent conditions. To obtain more information regarding the underlying processes we will use neurocognitive methods (EEG, fMRI) in future replications of this study.

Apart from the overall congruency effect, we observed stronger affective priming for congruent positive than congruent negative sentences: Sentences with positive adjectives after positive nouns were verified faster than sentences with positive adjectives after negative nouns. Mean verification times for sentences with negative adjectives after negative nouns were also shorter than those for sentences with negative adjectives after positive nouns, but this effect was smaller than the priming effect for congruent positive ones and was not significant. Such an unbalanced priming effect only for congruent positive sentences was neither suggested by the affective priming literature nor by the literature on supralexical consequences of emotional congruency. In both fields, congruent and incongruent trials usually are not differentiated with respect to the emotional connotation of the prime. We can reasonably well rule out the possibility that this unbalanced priming for positive words was due to a confound of the valence manipulation with the associative strength between nouns and adjectives. As described in the Methods Section, semantic association strength was kept constant across all conditions. One explanation for the observed unbalanced priming effect is suggested by the recently reported phasic affective modulation hypothesis of Topolinski and Deutsch (2013). This hypothesis rests upon the assumption that affect regulates the breadth or extent of spreading activation from a prime to close and remote semantic associates, with positive mood fostering semantic spread and negative mood inhibiting it (cf. Storbeck and Clore, 2008). Topolinski and Deutsch demonstrated that this affective modulation can be observed not only for modulation on a tonic temporal, but also on a phasic level. For example, the presentation of a positive tone or a positive face in one trial increased semantic priming particularly for weak associations even if the prime was presented simultaneously with the affect-inducing stimuli. They therefore concluded that an affective prime not only induced a spread of activation, but also a phasic affective modulation.

Thus, this phasic affective modulation might also play a role in the sentence verification paradigm. Sentences with positive nouns might induce a positive phasic mood modulation increasing spreading activation between semantic word units. This could lead to stronger affective priming for congruent positive sentences observed in our study. Still, this modulation effect should also at play in sentences with neutral adjectives. Semantic associations between positive and negative nouns and positive adjectives were as high as those between both noun types and neutral adjectives. If positive phasic modulation increased semantic priming, sentences with neutral adjectives after positive nouns should also be verified faster than sentences with neutral adjectives after negative nouns. However, we found no evidence for this. Thus, the phasic mood modulation hypothesis cannot fully account for the unbalanced priming effect for congruent positive sentences. This might be the case for all approaches focusing on mechanisms, which influence early processing stages like automatic spread of semantic activation. We assume that mechanisms at the supralexical level offer an alternative or complementing approach. In contrast to the standard affective priming paradigm, which does not require deeper semantic processing and especially integration of prime and target, the sentence verification task clearly requires the construction of a situation model to yield correct answers.

A promising account of the unbalanced priming effect is based on recently described valence effects related to the distribution and/or frequency of affective words and the *semantic cohesiveness hypothesis* (cf. Hofmann and Jacobs, 2014). Westbury et al. (2014) demonstrated for a very large corpus of affective words, that positive words are usually characterized by high frequencies. Negative words are more extreme on average in absolute valence magnitudes. The authors concluded that positive and negative words should be interpreted as distinct sets with possible differences on other dimensions than valence and frequency. The fact that negative events tend to be more finely differentiated than positive events is one example (Rozin and Royzman, 2001; Rozin et al., 2010) that is best illustrated in discrete emotion theories which since Darwin contain more negative than positive emotions. It could be assumed that finer differentiation of negative events hampers processing at the supralexical level, especially semantic integration and situation model construction: if negative words are less homogenous building coherent situation models for sentences with two negative words could be harder compared to sentences with two positive words. Recently, reported results according to which positive words provide a greater amount of semantic associations than negative words are in line with this account (Hofmann et al., 2011; Hofmann and Jacobs, 2014). For sentences with two positive words, semantic activation can spread across wider associative pathways, and thereby elicit a *positivity bias during meaning construction. First* evidence for this assumption is reported by Jacobs et al. (2015, this issue). In a study on the comprehension of affectively uni- and bi-valent noun-noun-compounds (NNCs), Jacobs et al. reported that comprehensibility ratings for NNCs containing two positive nouns were significantly higher than ratings for bipolar NNCs (containing both a positive and a negative noun), and also higher than univalent NNCs with two negative nouns. Based on the assumption that meaning construction is a necessary step for sentence verification, we thus would like to propose that the processing advantage observed for emotionally congruent sentences with two positive words also occurs at the supralexical level. Nevertheless, future studies are necessary to test this hypothesis directly.

In addition to the online processing of sentences, we were also interested in the emotional evaluation at the supralexical level, as expressed by sentence-level affective ratings. In general, our valence ratings indicated that the affective meanings of both nouns and adjectives contributed to the valence of the whole sentences, since we observed simple main effects for both. Sentences with negative nouns were rated more negatively than sentences with positive nouns. The same was observed for adjectives. Sentences with negative adjectives were rated more negatively than sentences with neutral adjectives, which were rated more negatively than sentences with positive adjectives. While these main effects are in line with the simple model predicting the emotion potential of supralexical units to be an algebraic sum of constituent word valences (Bestgen, 1994; Whissell, 1994), this is not the case for the observed strong interaction effect for the supralexical valence evaluations. Sentences with positive and neutral adjectives after negative nouns were evaluated more negatively than sentences with positive and neutral adjectives after positive nouns. For sentences with negative adjectives, no differences in supralexical valence ratings were observed. These sentences were generally evaluated as strongly negative, independently of whether they contained a positive or negative noun. These interactions are not in line with the simple model, but fit nicely with the assumed negativity bias especially for adjectives, described in the Hypothesis Section. The assumed dominance of emotional adjectives in the evaluation of supralexical units and the often reported negativity bias (Baumeister et al., 2001) predicted that supralexical valence ratings for sentences with negative adjectives are biased into a more negative direction. Our results demonstrated not only a simple bias into a more negative direction. Rather, negative adjectives dominated the supralexical valence evaluation, indicated by the fact that noun valence had no notable influence on the overall valence evaluation for sentences with negative adjectives. Our results for the supralexical valence ratings correspond to the results reported by Liu et al. (2013) who also observed a strong influence of negative adjectives especially on positive noun evaluation. Liu et al. discussed the general negativity bias as a main explanation for this effect. It is widely believed that negative stimuli attract more attention than positive ones (for review see Rozin and Royzman, 2001) and have a stronger influence on evaluation processes (Cacioppo et al., 1997; Ito et al., 1998). We thus propose, that, at least in part, the superiority effect for negative adjectives observed in this study can be explained with the negativity bias as well. Nevertheless, the fact that we observed the negativity bias only for negative adjectives points toward the importance of the specific syntactic role of adjectives. Normally, adjectives are used as noun phrase modifiers, a fact that underlines the relevance of adjectives for the meaning construction of whole sentences. But while this is true for all six conditions used in this study, a superiority effect was observed only for negative adjectives. For sentences with neutral and positive adjectives the valence of the noun still influenced the evaluation of the whole sentence. We therefore concluded that the observed superiority effect for negative adjectives indicated an interaction between the case role and the emotional valence of adjectives. Alternatively, the serial position of adjectives, which were presented always at the end of the sentences, could also be relevant. Further studies thus have to be conducted to fully explore the contributions of valence, syntactic role, and order for affective evaluations of supralexical units.

Besides the negativity bias for negative adjectives, we also observed a difference in the valence ratings for sentences with positive and neutral adjectives. The difference between the valence evaluations of sentences with positive and negative nouns was stronger for sentences with positive than for sentences with neutral adjectives. In other words, the condition in which the shortest verification times were observed, received the highest valence ratings. It can therefore be assumed that ease of processing and meaning construction are positively related to valence evaluation. This kind of relationship is discussed with regard to aesthetic responses and perceived beauty (Reber et al., 2004). The hedonic fluency hypothesis states that simply because a stimulus is processed faster or more fluently, it is accompanied by a positive affective evaluation leading to more positive aesthetic responses (Winkielman and Cacioppo, 2001; Reber et al., 2004). The results of two studies using pictorial stimuli are in line with this. Kuchinke et al. (2009) showed that the time to recognize a depicted object was shortest for high processing fluency paintings, which were also rated higher in their preference. Similarly, Albrecht and Corbon (2014) demonstrated especially for pictures with initially positive valence that highly fluent pictures were rated more positive than pictures of low processing fluency. For supralexical units, Bohrn et al. (2013) reported higher beauty ratings for familiar compared to unfamiliar and therefore harder-to-process German proverbs. Nevertheless, the same study demonstrated that the hedonic fluency hypothesis is insufficient to explain other effects observed in the processing of supralexical units, especially at the neuronal level. Hence, future studies should try to shed light on the interaction of processing fluency in encoding and meaning construction and the emotional evaluation of simple sentences and longer supralexical units.

## Conclusion

Our study introduces the sentence verification task as a paradigm for investigating the influence of emotional features of single words on the processing of supralexical units. We replicated both the processing advantage for positive compared to neutral and/or negative words often described in studies of single word processing, and an affective priming effect within a sentence context. Sentence verification was easiest for sentences containing emotionally congruent words with positive valences. We interpret this as a first evidence for easier semantic integration and situation model construction for sentences containing positive words. Sentence valence evaluations showed a negativity bias especially for negative adjectives indicating an interaction of the general negativity bias reported in the emotion processing literature, and the syntactic role of words during sentence processing. The comparison of verification times and valence evaluations pointed to an interrelationship between ease of processing, meaning construction, and affective evaluation, because easy-to-process sentences were rated more positive than harder-to-process sentences. Nevertheless, although we controlled for possible arousal effects in all our analyses, we could not fully exclude an independent effect of arousal as reported for example in Bayer et al. (2010). Further research should therefore manipulate both valence and arousal simultaneously.

Taken together, all emotional effects indicated that the comprehension of simple supralexical units is a highly interactive process (e.g., McClelland et al., 1989). If these results can be replicated also with more complex sentences it would seriously challenge future models of sentence comprehension and text processing to go beyond “cold” information processing and include “hot” affective and aesthetic processes, and provide stronger constraints for very general frameworks like the neurocognitive poetics model of literary reading which includes such processes (Jacobs, 2011, 2015a,b). The model has received empirical support at the experiential, behavioral and neuronal levels for word, phrase, poem, and story processing (Altmann et al., 2012; Bohrn et al., 2013; Hsu et al., 2014, 2015a,b,c; Lüdtke et al., 2014; Aryani et al., 2015; Jacobs, 2015b; Lehne et al., 2015) but still is underspecified for predicting effects in tasks like sentence verification.

### Conflict of interest statement

The authors declare that the research was conducted in the absence of any commercial or financial relationships that could be construed as a potential conflict of interest.

## Appendix

### (A) The lmer specification for the model predicting verification times

> library (lme4)

> library (car)

> contrasts (data$VG-A) = contr.sum(3)

> contrasts (data$VG-N) = contr.sum(2)

> LMM_RT = lmer (1/RT ~ 1 + VG-N ^*^ VG-A + cARO-N + cARO-A + (1 |Subject) + (1 + VG-N + VG-A |Itemset), data, REML = T)

> summary (LMM_RT)

> Anova (LMM_RT, test = “F”)

### (B) The lmer specification for the model predicting valence ratings

> contrasts (data$VG-A) = contr.sum(3)

> contrasts (data$VG-N) = contr.sum(2)

> LMM_VR = lmer (sqrt(Valence_Ratings) ~ 1 + VG-N ^*^ VG-A + cARO-N + cARO-A + (1 + VG-A |Subject) + (1 + VG-N ^*^ VG-A |Itemset), data, REML = T)

> summary (LMM_VR)

> Anova (LMM_VR, test = “F”)
